# Selective inhibition of TRPV3 channel by natural rosmarinic acid and its analogs for alleviation of skin lesions through downregulation of NF-κB pathway

**DOI:** 10.1016/j.jbc.2025.110267

**Published:** 2025-05-21

**Authors:** Yaxuan Qu, Shilun Mo, Fei Hou, Ningning Wei, Xiaoying Sun, KeWei Wang

**Affiliations:** 1 Department of Pharmacology and, School of Pharmacy, Qingdao University Qingdao Medical College, Qingdao, China; 2 Natural Medicinal Chemistry and Pharmacognosy, School of Pharmacy, Qingdao University Qingdao Medical College, Qingdao, China; 3 Department of Pharmacy, the Affiliated Hospital of Qingdao University, Qingdao, China; 4 Institute of Innovative Drugs, Qingdao University, Qingdao, China

**Keywords:** carvacrol, dermatitis, NF-κB, NF-κB inhibitor, rosmarinic acid, rosamarinic analogs, TRPV3

## Abstract

Topical application of natural phenolic compound rosmarinic acid (ROSA) and its analogs is known to exert pharmacological effects, including anti-inflammation, antiallergy, and antioxidant properties. However, the mechanism of action of the ROSA remains largely unknown. Here, we describe a novel role of natural phenolic ROSA and its analogs in the selective inhibition of warmth-sensitive Ca^2+^-permeable cutaneous TRPV3 channel for the alleviation of skin lesions through the downregulation of NF-κB pathway. ROSA and its analogs, (E)-3-(3,4-dihydroxyphenyl)-N-(2-(3,4-dihydroxyphenyl) ethyl)-2-propenamide and methyl rosmarinate, inhibit macroscopic TRPV3 currents in both concentration-dependent and structure-dependent manners with IC_50_ values ranging from 10 to 160 μM. ROSA also directly inhibits single TRPV3 channels by reducing the channel open probability without altering its unitary conductance. ROSA selectively targets TRPV3 over other subtypes of thermos-TRPs such as TRPV1, TRPV4, TRPA1 and TRPM8 channels. Site-directed mutagenesis combined with molecular docking reveals two residues T636 and T665 critical for ROSA-mediated inhibition of TRPV3. Furthermore, network pharmacology identified downstream *p*-P65, TNF-α, and interleukin-6 proteins in NF-κB signaling pathway as critically involved in ROSA-mediated reduction of cell death and alleviation of skin lesions. Altogether, our findings demonstrate that ROSA exerts its anti-inflammatory effects by selectively inhibiting TRPV3 channel and suppressing the NF-κB signaling pathway.

Rosmarinic acid (ROSA) is a natural water-soluble caffeoyl ester polyphenol hydroxy compound found in various plants, particularly in herbs such as rosemary, basil, and oregano (1). ROSA and its analogs have been investigated for their diverse biological activities, including anti-inflammatory, anti-immunoresponse and antiphotoaging effects (2, 3, 4, 5, 6, 7). They have been shown to downregulate interleukin (IL)-1β, IL-6, IL-8, TNF-α, and other inflammatory factors in keratinocytes to inhibit the inflammatory reactions (8). Clinical studies on atopic dermatitis (AD) patients have demonstrated that the application of ROSA effectively alleviates skin water loss, erythema, itching, and improves skin condition (9). ROSA exhibits protective effects against oxidative stress, cell apoptosis and DNA damage and skin tumors (2, 6, 10). In addition to its effects on the skin, ROSA also shows promise in managing pain, inflammatory bowel disease, arthritis, asthma, allergic rhinitis, and other disease conditions (5, 11). Despite numerous experimental studies demonstrating the protective effects of ROSA and its analogs on the skin, their direct targets have yet to be elucidated. Therefore, it is of great importance to explore the direct targets of ROSA for future clinical applications.

The skin serves as the first body’s primary barrier against external threats, and its homeostasis is crucial in safeguarding against damage. Skin damage or dysfunction of keratinocytes are primary causes and exacerbating factors in various skin diseases such as wound repair, dermatitis, itching, dryness, and photoaging (12). Calcium signaling plays a pivotal role in numerous pathological and physiological processes, affecting cell life cycles of keratinocytes in the skin (13). As a key factor in the differentiation and proliferation of keratinocytes, changes in the Ca^2+^ concentration gradient impact the vertical differentiation process from the basal layer to the stratum corneum (14). Intracellular Ca^2+^ assists wound healing and contributes to barrier and intercellular junction formation by promoting cell extension and migration (15). Additionally, Ca^2+^ regulates hyaluronic acid metabolism, lipid synthesis, and secretion, maintains epidermal barrier osmotic stability, and induces cell apoptosis (16). Moreover, the crosstalk between Ca^2+^ and reactive oxygen species is considered a significant factor in the body’s oxidative stress inflammatory response (17). Therefore, both extracellular and intracellular Ca^2+^ signaling plays vital roles in epidermal balance and homeostasis.

An increasing number of studies have demonstrated that various TRP channels play significant roles in regulating skin itching, inflammation, and other diseases, thus making them prospective therapeutic targets (18). Gain-of-function mutations of TRPV3 in keratinocytes are closely associated with skin barrier imbalance, wound healing, pruritus, dermatitis, alopecia, and genetic Olmsted syndrome (OS) (19, 20, 21, 22, 23). Overactive TRPV3 in rodents induces phenotypes akin to AD and alopecia (24, 25). Activation of TRPV3 has been shown to inhibit hair growth by suppressing the proliferation of outer root sheath cells (26). In mice with TRPV3 G568V point mutation, excessive activation of TRPV3 damages hair follicles and shafts, induces hair follicle cell apoptosis, and impedes normal hair growth, whereas topical inhibition of TRPV3 promotes hair growth (19). Inhibition of TRPV3 also downregulates the expression of inflammatory factors and alleviates AD-like conditions induced by compounds such as 2,4-dinitrofluorobenzene, carvacrol (Car) and MC903 in various animal models (27, 28, 29). Our recent research has demonstrated that pharmacological or genetic inhibition of TRPV3 can alleviate UVB-induced ear swelling (30). Although the role of TRPV3 in skin related diseases has been increasingly explored, selective TRPV3 inhibitors still remain scarce. We have reported several selective TRPV3 inhibitors, including forsythiaside B, verbascoside, and isochlorogenic acid A/B (28, 31, 32). Interestingly, the structure of ROSA shares the same cinnamate ester skeleton as these inhibitors. The cinnamate skeleton has been identified as an active group of TRPV3 inhibitors (33). Therefore, considering the important role of ROSA in skin diseases and its structural characteristics, we hypothesize that ROSA likely acts on the TRPV3 channel.

In this study, we investigated the inhibitory effects of ROSA and its analogs on TRPV3 and NF-κB signaling pathway. Our findings showed that ROSA as a selective inhibitor of TRPV3 mitigates skin lesions induced by the agonist skin sensitizer Car at both *in vitro* and *in vivo* levels. Our data demonstrated that inhibition of TRPV3 by ROSA exerts its anti-inflammatory effects and downregulates the NF-κB signaling.

## Results

### ROSA inhibits macroscopic and single-channel TRPV3 currents

**Figure 1:**
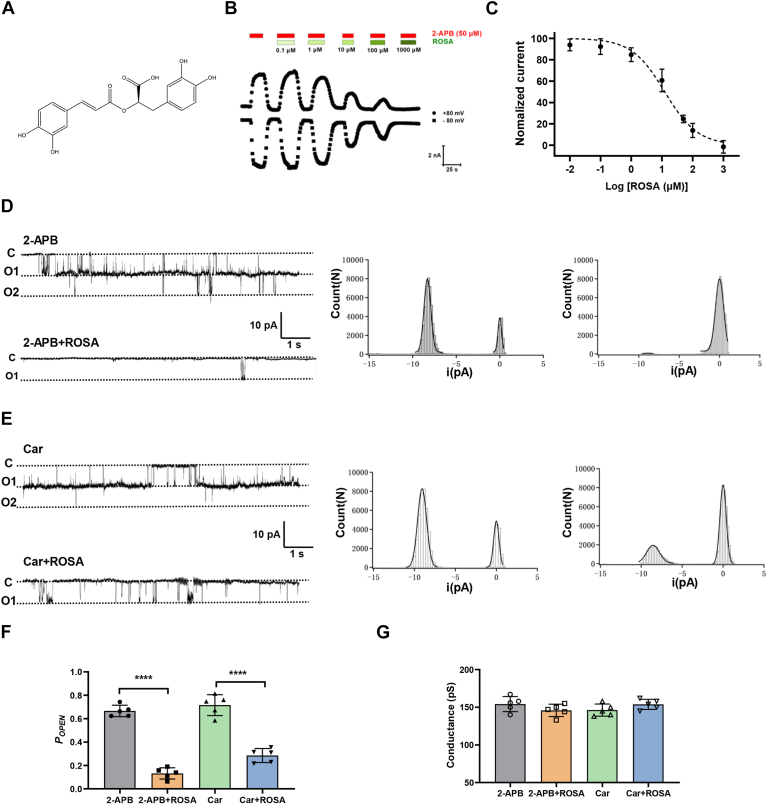
**Concentration-dependent inhibition of whole-cell hTRPV3 currents and inhibition of single hTRPV3 channel currents in HEK293T cells by natural ROSA.***A*, chemical structure of ROSA. *B*, inhibition of whole-cell currents by increasing concentrations of ROSA at 0.1 to 1000 μM. *C*, analysis of concentration-dependent inhibition of hTRPV3 outward currents at +80 mV by ROSA with an IC_50_ value of 12.4 ± 4.2 μM. *D*, *left panels*: representative traces recorded from an inside-out patch in the condition of after addition of 50 μM 2-APB, co-perfusion of 100 μM ROSA, and 50 μM 2-APB. The *middle and right panels*, all-points histograms of single-channel current recording data as shown in *leftpanels*, and their histograms were fitted to a Gauss functions. *E*, *left panels*: representative traces recorded from an inside-out patch in the condition of after addition of 300 μM carvacrol (Car), co-perfusion of 100 μM ROSA, and 300 μM carvacrol. The *middle and right panels*, all-points histograms of single-channel current recording data as shown in *upper panels*, and their histograms were fitted to a Gauss functions. *F*, summary for calculated single hTRPV3 channel mean *P*_*OPEN*_ values in the presence of different treatments of (*D*) and (*E*) mentioned above (n = 5, ∗∗∗∗*p* < 0.0001, by one-way ANOVA, followed by the Tukey’s test). *G*, summary of hTRPV3 single-channel conductance after exposure to different treatments of (*D*) and (*E*) mentioned above (n = 5). Data are expressed as the mean ± SD. 2-APB, 2-aminoethoxydiphenyl borate; ROSA, rosmarinic acid.

We carried out recordings of macroscopic whole-cell hTRPV3 channel currents expressed in Human embryonic kidney (HEK) 293T cells and tested the effect of ROSA on the channel currents activated by agonist 2-aminoethoxydiphenyl borate (2-APB) (50 μM) at different concentrations ranging from 0.01 to 1000 μM on the channel currents (Fig. 1, *A* and *B*). ROSA inhibited hTRPV3 currents in a dose-dependent manner with an IC_50_ value of 12.4 ± 4.2 μM (Fig. 1, *B* and *C*).

To confirm ROSA directly acting on hTRPV3, we further recorded the single-channel currents in an inside-out patch configuration. Perfusion of TRPV3 agonist 2-APB (50 μM) or Car (300 μM) led to the single-channel openings with the open probability of 0.67 ± 0.05 and 0.77 ± 0.09 (Fig. 1, *D*–*F*). In contrast, co-perfusion of ROSA (100 μM) with either 2-APB (50 μM) or Car (300 μM) reduced the channel openings, resulting in a decreased open probability of 0.13 ± 0.05 and 0.28 ± 0.06, respectively, without altering the channel unitary conductance (Fig. 1, *D*–*G*). These results indicate that ROSA inhibits macroscopic TRPV3 currents in a concentration-dependent manner by reducing the single-channel open probability.

### Selective inhibition of TRPV3 currents by ROSA over other subtypes of TRP channels

**Figure 2:**
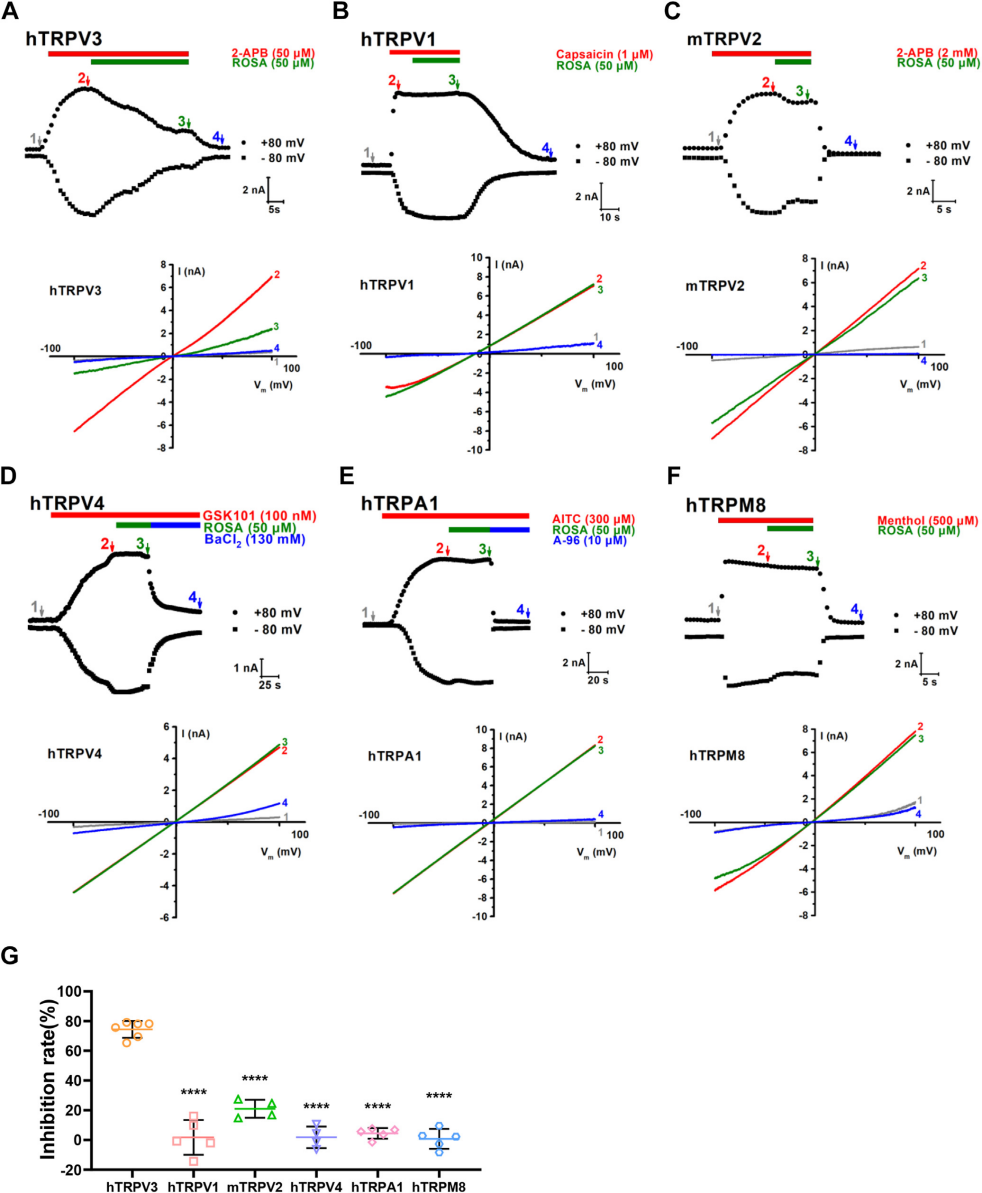
**Selective inhibition of whole-cell hTRPV3 currents by ROSA over other thermoTRP channels transiently expressed in HEK293T cells.***A*–*F*, *upper panels*: whole-cell currents of hTRPV3 in response to 50 μM 2-APB and co-application of 50 μM ROSA, hTRPV1 in response to 1 μM capsaicin and co-application of 50 μM ROSA, mTRPV2 in response to 2 mM 2-APB and co-application of 50 μM ROSA, hTRPV4 in response to 100 nM GSK1016790A and co-application of 50 μM ROSA, hTRPA1 in response to 300 μM AITC and co-application of 50 μM ROSA, and hTRPM8 in response to 500 μM menthol and co-application of 50 μM ROSA. *Bottom panels*: current-voltage curves of TRP channel in response to voltage ramps from −100 mV to +100 mV from the above panel before (1) and after channel agonist (2), after coaddition of 50 μM ROSA and channel agonist (3), and after washout or inhibition (4). *G*, summary for average current inhibition of hTRPV3, mTRPV2, hTRPV1, hTRPV4, hTRPA1, and hTRPM8 channels by 50 μM ROSA (∗∗∗∗*p* < 0.0001, compared with hTRPV3, by one-way ANOVA, followed by the Dunnet’s test). Data are expressed as the mean ± SD. 2-APB, 2-aminoethoxydiphenyl borate; AITC, allyl isothiocyanate; ROSA, rosmarinic acid.

To determine if ROSA selectively inhibited TRPV3 channels, we tested the effect of ROSA on several other TRP subtypes, including hTRPV1, hTRPV4, hTRPA1, hTRPM8, and mTRPV2 currents that were activated by their agonists, capsaicin (1 μM), GSK101 (100 nM), allyl isothiocyanate (300 μM), menthol (30 μM), and 2-APB (2 mM), respectively. As shown in Figure 2, *A*–*F*, ROSA at 50 μM inhibited TRPV3 currents by approximately 74.3 ± 5.7%. In comparison, ROSA exhibited little inhibition for TRPV1 (1.8 ± 11.7%), TRPV4 (2.0 ± 7.3%), TRPA1 (4.5 ± 3.6%), and TRPM8 (0.7 ± 6.7%) or weak inhibition for TRPV2 (21.0 ± 6.1%) (Fig. 2*G*). These results indicate that ROSA selectively inhibits TRPV3 channels over other tested subtypes of TRP channels.

### Identification of residues critical for ROSA and its analogs binding to TRPV3

**Figure 3:**
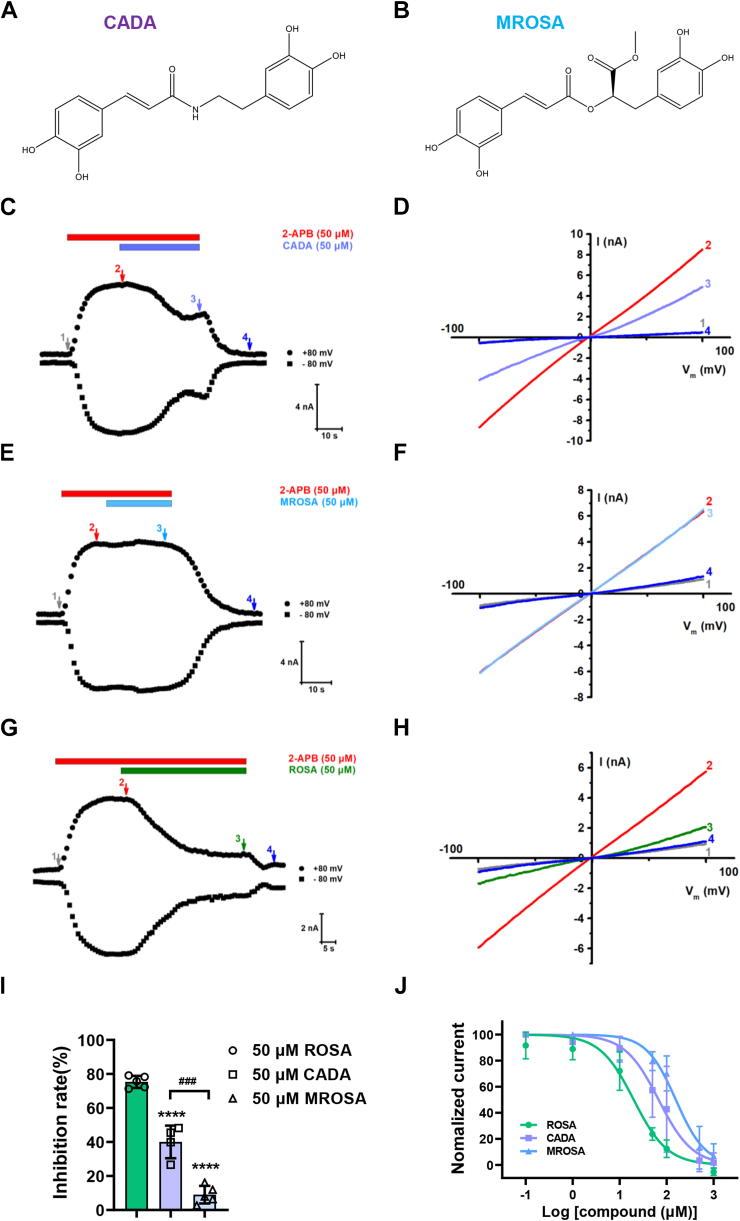
**Concentration-dependent inhibition of hTRPV3 currents by ROSA analogs CADA and MROSA.***A* and *B*, chemical structures of CADA and MROSA. *C*, *E*, and *G*, whole-cell currents of hTRPV3 in response to 50 μM 2-APB and co-application of 50 μM CADA, 50 μM MROSA, or 50 μM ROSA. *D*, *F*, and *H*, current-voltage curves of hTRPV3 channel in response to voltage ramps from −100 mV to +100 mV from the *left panel* before (1) and after 50 μM 2-APB (2), after coaddition of 50 μM CADA, MROSA, or ROSA and 50 μM 2-APB (3), and after washout (4) corresponding to (*C*), (*E*), and (*G*). *I*, summary for average current inhibition of hTRPV3 by 50 μM ROSA, 50 μM CADA, and 50 μM MROSA (n = 4–5, ∗∗∗∗*p* < 0.0001, compared with 50 μM ROSA, by one-way ANOVA, followed by the Dunnet’s test, ^###^*p* < 0.001, compared with 50 μM MROSA, by unpaired *t* test). *J*, analysis of concentration-dependent inhibition of hTRPV3 outward currents at +80 mV by ROSA with an IC_50_ value of 20.0 ± 4.5 μM, CADA with an IC_50_ value of 67.7 ± 22.4 μM, and MROSA with an IC_50_ value of 157.1 ± 34.5 μM (n = 5–7). Data are expressed as the mean ± SD. 2-APB, 2-aminoethoxydiphenyl borate; MROSA, methyl rosmarinate; ROSA, rosmarinic acid.

To identify residues critical for ROSA binding to TRPV3, we further investigated the inhibitory effects of ROSA and its two structure analogs, (E)-3-(3,4-dihydroxyphenyl)-N-(2-(3,4-dihydroxyphenyl) ethyl)-2-propenamide (CADA) (Fig. 3*A*) and methyl rosmarinate (MROSA) (Fig. 3*B*) on TRPV3 currents. As shown in Figure 3, *C*–*I*, ROSA at 50 μM inhibited TRPV3 by approximately 75.3 ± 3.5%, while its analog CADA inhibited it by about 40.1 ± 9.6% and MROSA by only 9.0 ± 5.2%. Perfusion of CADA and MROSA resulted in dose-dependent inhibition effects of hTRPV3 currents evoked by 2-APB (50 μM) with significant reduction of potencies and IC_50_ values of 67.7 ± 22.4 μM and 157.1 ± 34.5 μM, respectively (Fig. 3*J*). These results demonstrate that ROSA and its analogs CADA and MROSA inhibit TRPV3 currents in their structure-dependent manner.

**Figure 4:**
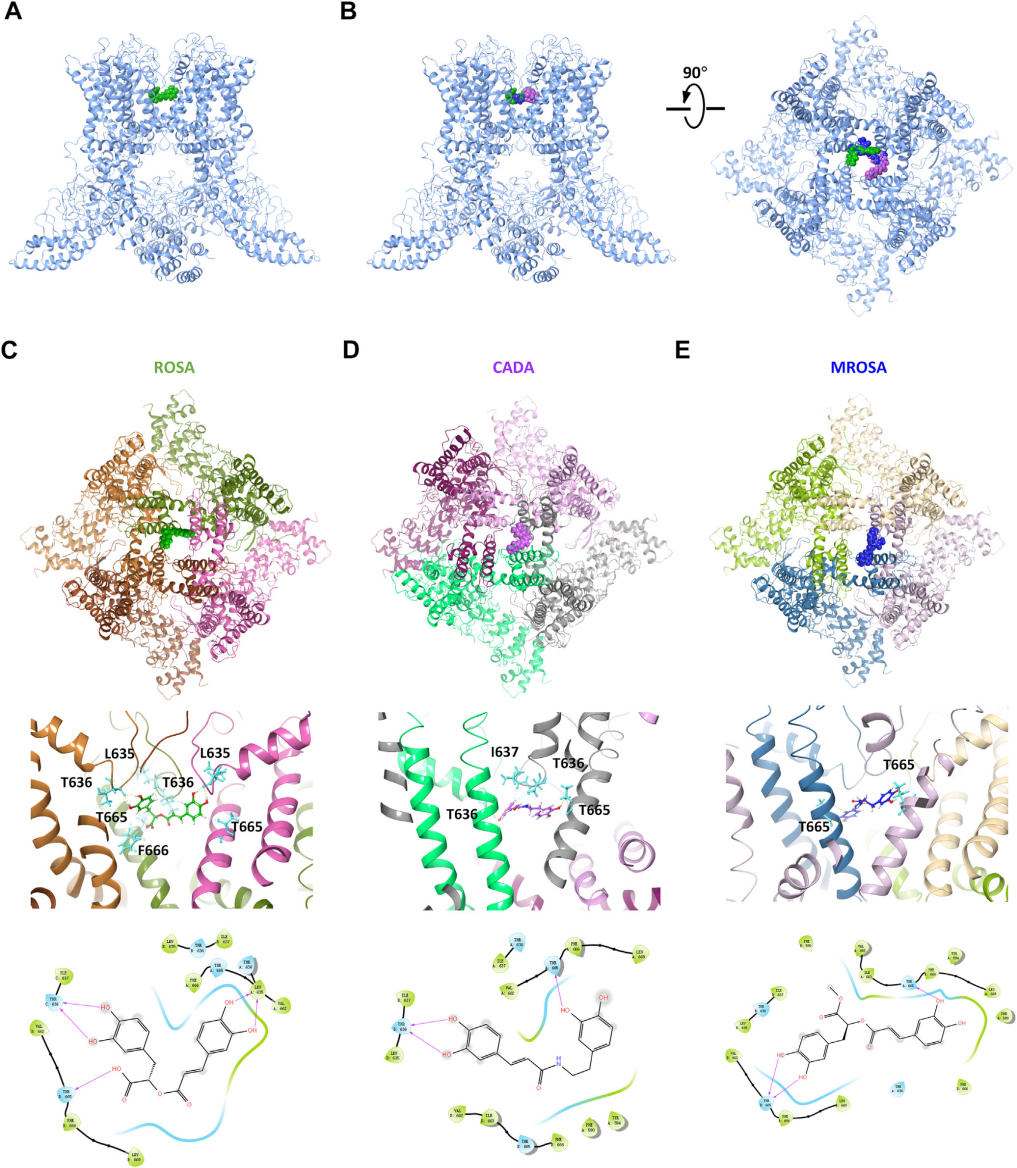
**Molecular docking for binding sites for ROSA and its analogs in TRPV3 channel.***A*, representative bound conformations of ROSA confined to the pocket consisting of the pore helix and S6 segment in *side view*. *B*, representative bound conformations of ROSA, CADA, and MROSA confined to the pocket consisting of the pore helix and S6 segment in *side view* (*left*) and *top-down view* (*right*). ROSA, CADA, and MROSA are shown as *green*, *purple*, and *blue*, respectively. *C*–*E*, *upper panels*: representative bound conformations of ROSA CADA or MROSA confined to the pocket consisting of the pore helix and S6 segment in *top-down view*. *Middle panels*: *side view* of ROSA (*green*), CADA (*purple*), or MROSA (*blue*) and mTRPV3 subunit (PDB: 6DVW) interactions. *Lower panels*: 2D interaction diagram corresponding above panel. MROSA, methyl rosmarinate; ROSA, rosmarinic acid.

We further performed molecular docking of ROSA, and its analogs (CADA and MROSA) onto the cryo-EM structure of mTRPV3 (PDB: 6DVW) using Schrödinger. The docking predicted that ROSA, CADA, and MROSA are confined near the selective filter (S5-S6 linker and S6) (Fig. 4, *A* and *B*). ROSA is mainly recognized by four residues L635, T636, T665, and F666 with a binding score of −6.578 (Fig. 4*C*). CADA is recognized by similar three residues T636, I637, and T665 with a binding score of −6.526 (Fig. 4*D*), while MROSA is only recognized by one residue, T665, with a binding score of −6.120 (Fig. 4*E*).

**Figure 5:**
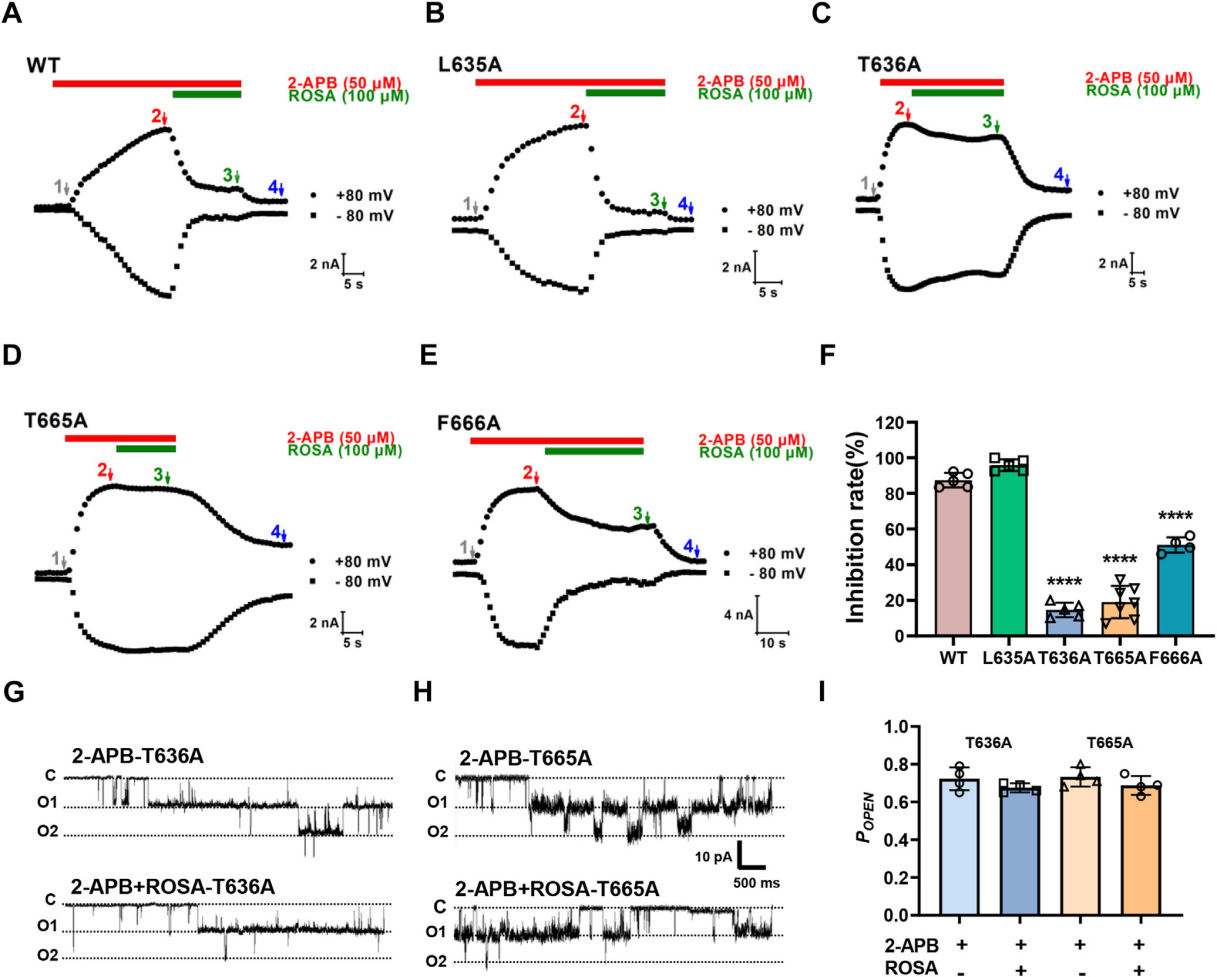
**Identification of key sites residues for ROSA binding to TRPV3 channels.***A*–*E*, representative current traces of WT hTRPV3, L635A, T636A, T665A, and F666A mutants expressed in HEK293T cells in responses to 2-APB alone and 2-APB with 100 μM ROSA. *F*, summary for WT hTRPV3 and mutant current inhibition by 100 μM ROSA (n = 5–7, ∗∗∗∗*p* < 0.0001, compared with WT group, by one-way ANOVA, followed by the Dunnet’s test). *G* and *H*, representative traces recorded from an inside-out patch in the condition of 50 μM 2-APB, co-perfusion of 100 μM ROSA, and 50 μM 2-APB in T636A or T665A mutants expressed in HEK293T cells. *I*, summary for calculated single hTRPV3 mutants, T636A and T665A, mean *P*_*OPEN*_ values in the presence of 50 μM 2-APB and co-application of 100 μM ROSA (n = 4, by paired *t* test). Data are shown as the mean ± SD. 2-APB, 2-aminoethoxydiphenyl borate; ROSA, rosmarinic acid.

To further confirm those residues critical for ROSA binding to TRPV3, we generated four alanine mutations: L635A, T636A, T665A, and F666A. As shown in Figure 5, *A*–*F*, mutating two residues T636A and T665A individually markedly reduced ROSA-induced inhibition of macroscopic hTRPV3 currents to 14.7% and 19.1%, respectively, as comparted with 87% inhibition for the WT hTRPV3 currents. We also tested the effects of ROSA on the single-channel currents of the two mutants TRPV3 T636A and TRPV3 T665A. ROSA exhibited little reduction of single-channel open probability with 0.67 and 0.69 the T636A and T665A mutants, respectively (Fig. 5, *G*–*I*). These results demonstrate that the TRPV3 residues T636 and T665 are critical for interacting with ROSA.

### ROSA exerts anti-inflammatory effects on ear swelling and dermatitis *via* inhibiting TRPV3

**Figure 6:**
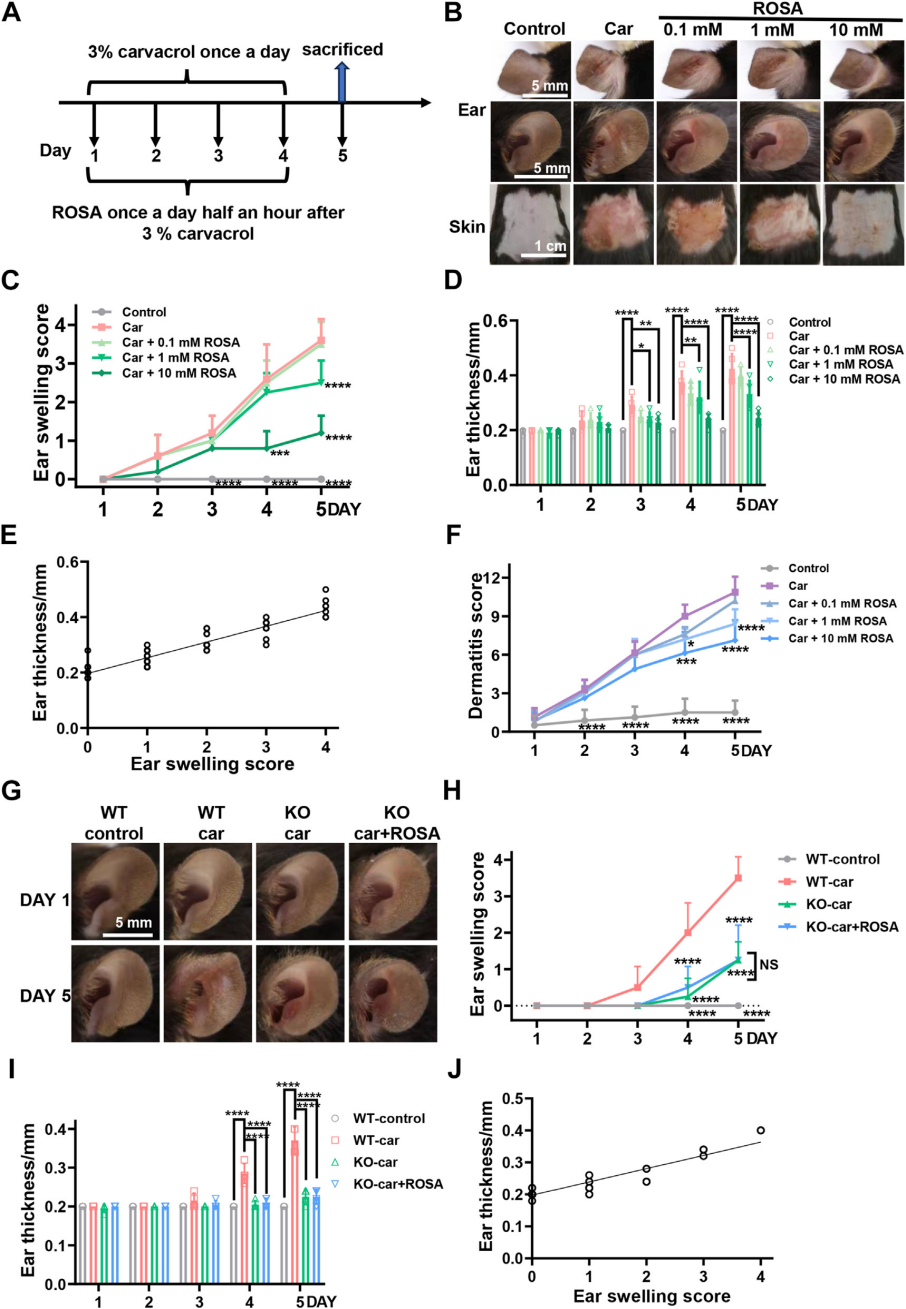
**Time- and dose-dependent alleviation of carvacrol-induced ear and dorsal skin inflammation by ROSA.***A*, a flowchart of carvacrol-induced ear swelling in WT mice and *Trpv3*-KO mice treated with topical ROSA for four consecutive days. Topical 3% carvacrol in 100 μl was applied to the ear once for ear swelling. Ten millimolars ROSA in 100 μl were topically applied once a day half an hour after applying carvacrol for four consecutive days. *B*, phenotypic observation of mouse ear and dorsal skin at day 5 in the groups of solvent control, 3% carvacrol alone, and 3% carvacrol with ROSA at 0.1 mM, 1 mM, and 10 mM. *C*, ear swelling scores of mice treated with or without carvacrol and ROSA (n = 5, ∗∗∗*p* < 0.001, ∗∗∗∗*p* < 0.0001). *D*, summary of ear thickness for carvacrol-induced ear swelling in mice treated with or without carvacrol and ROSA (n = 5, ∗*p* < 0.05, ∗∗*p* < 0.01, and ∗∗∗∗*p* < 0.0001). *E*, scatter plot illustrating the correlation between the ear thickness and ear swelling scores from Figure 6, *C* and *D* (n = 5, Spearman coefficient method, r = 0.8904; *p* < 0.0001, two-tailed, Gaussian approximation, α = 0.05). *F*, dermatitis scores of mice in different groups treated with or without different concentrations of ROSA for five consecutive days (n = 5, ∗*p* < 0.05, ∗∗∗*p* < 0.001, and ∗∗∗∗*p* < 0.0001, by two-way ANOVA, followed by the Dunnet’s test). *G*, representative images for mouse ear swelling at day 1 and day 5 before and after topical treatment. *H*, ear swelling scores of mice in WT or *Trpv3*-KO mice treated with or without carvacrol and ROSA (n = 4, NS, no significance, ∗∗∗∗*p* < 0.0001). *I*, summary of ear thickness for carvacrol-induced ear swelling in mice treated with or without carvacrol and ROSA (n = 4, ∗∗∗∗*p* < 0.0001, by two-way ANOVA, followed by the Dunnet’s test. *J*, scatter plot illustrating the correlation between the ear thickness and ear swelling scores from Figure 6, *H* and *I* (n = 4, Spearman coefficient method, r = 0.9054; *p* < 0.0001, two-tailed, Gaussian approximation, α = 0.05). Data are expressed as the mean ± SD. ROSA, rosmarinic acid.

To assess the anti-inflammatory effects of ROSA, we established a mouse model of ear swelling and dorsal skin inflammation induced by the skin sensitizer Car (3%) (Fig. 6*A*). We tested the anti-inflammatory effects of topical ROSA on both ear and dorsal skin at different concentrations (0.1, 1.0 and 10 mM). Topical ROSA significantly alleviated the ear and dorsal skin inflammation and reduced the ear swelling scores in both dose- and time-dependent manner, compared to the group treated with Car alone (Fig. 6, *B* and *C*). The reduction in ear thickness was also evident, starting on the third day of ROSA application, compared to the group treated with Car alone (Fig. 6*D*). The ear thickness was also positively correlated with its swelling scores (Fig. 6*E*). Similarly, dorsal skin inflammation showed a significant difference in dermatitis scores between the ROSA group and the Car group on the fourth day (Fig. 6*F*). These results demonstrate that ROSA reduces inflammation in both dose- and time-dependent manners *via* inhibiting TRPV3.

To further confirm the role of ROSA in reducing inflammation *via* TRPV3 inhibition, we tested the effect of ROSA in WT and *Trpv3* KO mice. Topical applications of Car for consecutive 4 days in WT mice resulted in ear skin inflammation with significantly increased thickness and ear swelling scores, as compared to *Trpv3* KO mice exhibiting a significant reduction of skin thickness and ear swelling scores (Fig. 6, *G*–*I*). Similarly, topical application of ROSA (10 mM) showed no further reduction of ear thickness in *Trpv3* KO mice (Fig. 6, *G*–*I*). The ear thickness was positively correlated with its swelling scores (Fig. 6*J*). The results demonstrate that ROSA-mediated alleviation of ear skin inflammation is dependent on TRPV3 inhibition.

### ROSA blocks the NF-κB signaling thorough downregulating its transcription factor *p*-P65 and inflammatory factors

**Figure 7:**
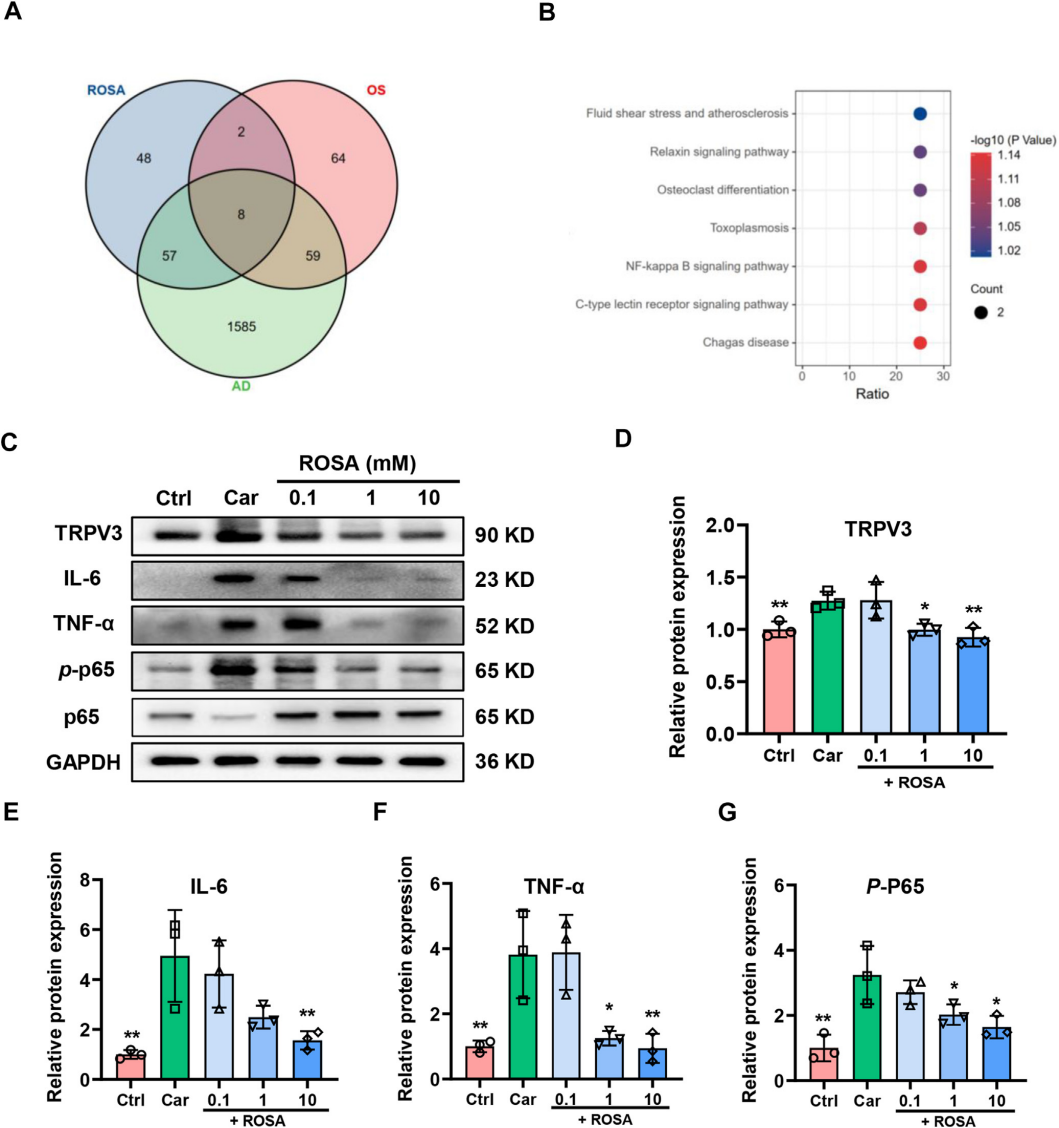
**ROSA blocks NF-κB activation by downregulating transcription factor *p*-p65 and inflammatory factors.***A*, a Venn diagrams elucidating three sets of databases associated with ROSA effects, gain-of-function TRPV3 mutations-associated Olmsted syndrome and atopic dermatitis (AD). *B*, identification of potential targets for KEGG pathway enrichment analysis of ROSA effects, gain-of-function TRPV3 mutations-associated Olmsted syndrome and atopic dermatitis (AD). *C*, protein expressions of TRPV3, IL-6, TNF-α, *p*-P65, P65, and GAPDH in mouse ear tissues on day 5 in response to different concentrations of ROSA at 0.1, 1, and 10 mM. *D*–*G*, analysis of protein expression levels of TRPV3, IL-6, TNF-α, and *p*-P65 in lysates of mouse ear tissues on day 5 (n = 3, ∗*p* < 0.05, ∗∗*p* < 0.01, compared with Car group, by one-way ANOVA, followed by Dunnett's multiple comparisons test). Data are expressed as the mean ± SD. Car, carvacrol; Ctrl, control; KEGG, Kyoto Encyclopedia of Genes and Genomes; ROSA, rosmarinic acid.

To understand the mechanism underlying the ROSA-mediated anti-inflammatory effects, we adopted network pharmacology approach and analyzed three databases of ROSA effects, gain-of-function TRPV3 mutations-associated OS and TRPV3-associated AD, and identified eight key targets, such as TNF, TGFB1, PTGS2, and MMP9, involved in ROSA-mediated alleviation of skin inflammation *via* inhibition of TRPV3 (Fig. 7*A*). The Kyoto Encyclopedia of Genes and Genomes pathway enrichment analysis revealed NF-κB signaling pathways critically involved in the ROSA-mediated effects (Fig. 7*B*). To gain further insights, we detected the protein expressions of TRPV3, key inflammatory factors of downstream NF-κB pathway in mouse skin tissue samples. We found that the expressions of TRPV3, IL-6, TNF-α, and *p*-P65 were upregulated in mouse ear tissues treated with 3% Car (Fig. 7, *C*–*G*). In contrast, topical applications of ROSA downregulated the upregulation of TRPV3, IL-6, TNF-α, and *p*-P65 proteins expressions induced by Car in dose-dependent manner (Fig. 7, *C*–*G*).

### Blockade of NF-κB signaling by inhibitor JSH-23 reduces cell death and dermatitis induced by overactive TRPV3 function

**Figure 8:**
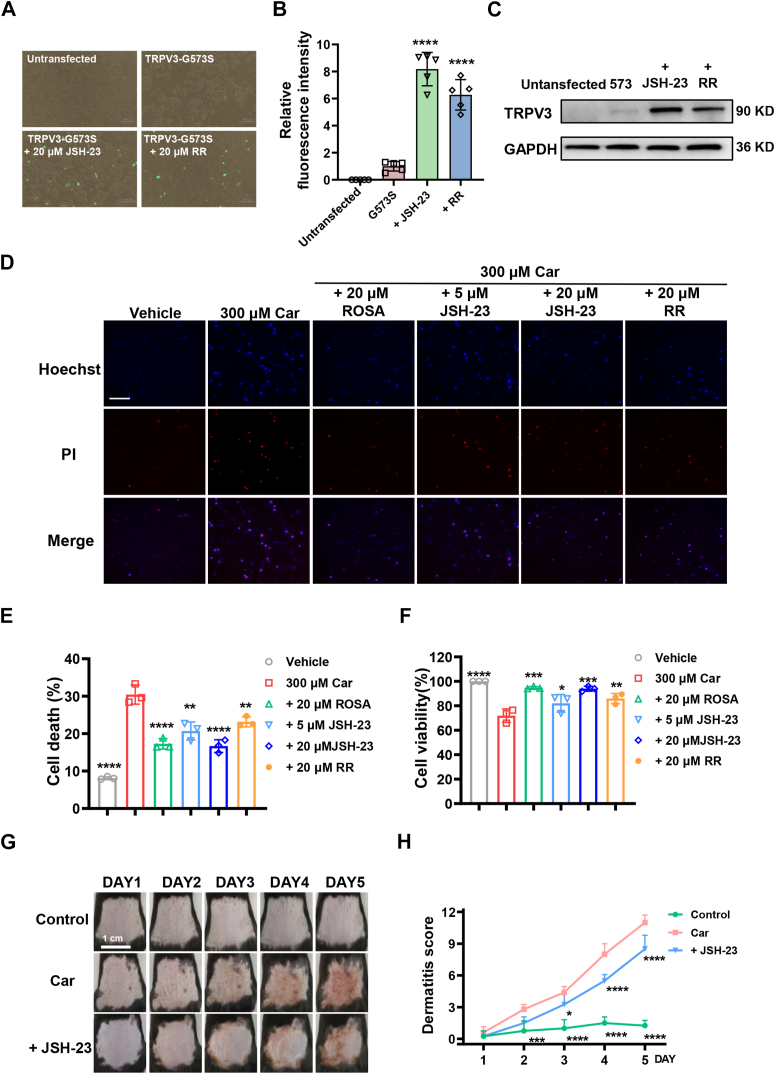
**Suppression of NF-κB signaling by JSH-23 reduces cell death and dermatitis induced by gain-of-function TRPV3 G573S mutation or carvacrol-mediated TRPV3 overactivation.***A*, representative fluorescence images of HEK293T cells with or without treatment expressing G573S-TRPV3 or not. *B*, summary for relative fluorescence intensity from (*A*) (n = 5, ∗∗∗∗*p* < 0.0001, compared with G573S group, by one-way ANOVA, followed by the Dunnet’s test). *C*, protein expressions of TRPV3 and GAPDH in HEK293T cells expressing G573S-TRPV3 or not in response to different treatments. *D*, representative fluorescence images stained with Hoechst and PI of HEK293T cells expressing hTRPV3 after carvacrol (300 μM) treatment for 1 h in the absence and presence of ROSA (20 μM), JSH-23 (5/20 μM), or ruthenium red (RR, 20 μM). *E*, summary for cell death ratio from (*D*) (n = 3, ∗∗*p* < 0.01, ∗∗∗∗*p* < 0.0001, by one-way ANOVA, followed by the Dunnet’s test). *F*, cell viability of HEK293T cells expressing hTRPV3 after carvacrol (300 μM) treatment with or without ROSA, JSH-23, and RR for 1 h in CCK-8 assay. *G*, representative phenotypic observation of mouse dorsal skin for five consecutive days in the groups of solvent control, 3% carvacrol alone, and 3% carvacrol with JSH-23. *H*, dermatitis scores of mice in different groups treated with or without Car and JSH-23 for five consecutive days (n = 4–5, ∗*p* < 0.05, ∗∗*p* < 0.01, ∗∗∗*p* < 0.001, and ∗∗∗∗*p* < 0.0001, by two-way ANOVA, followed by the Dunnet’s test). Data are expressed as the mean ± SD. CCK-8, cell counting kit-8; HEK, Human embryonic kidney; PI, propidium iodide; ROSA, rosmarinic acid.

Overactivation or gain-of-function mutation of TRPV3 can induce Ca^2+^ influx leading to lytic cell death and disruption of skin immune homeostasis (34). To confirm if the downstream NF-κB pathway involved in the cell death induced by overactive TRPV3 function, we tested the effect of NF-κB *p*-P65 inhibitor JSH-23 (20 μM) on cell death of HEK293T cells transfected with gain-of-function mutant GFP-TRPV3-G573S. Due to the presence of GFP fragments in TRPV3 plasmid, live HEK293T cells expressing TRPV3 proteins are shown in green. As shown in Figure 8, *A* and *B*, no green fluorescence was observed in untransfected-HEK293T cells, and almost no green fluorescence was observed in HEK293T cells expressing G573S-TRPV3, while live cells treated with NF-κB *p*-P65 inhibitor JSH-23 (20 μM) and ruthenium red (RR, 20 μM) exhibited robust green fluorescence, in which TRPV3 protein expressions in HEK293T cells were further confirmed in Western blot assay (Fig. 8*C*).

In addition, we also test the effect of ROSA (20 μM) and also NF-κB *p*-P65 inhibitor JSH-23 (5 μM or 20 μM, IC_50_ = 7.1 μM) on cell death of HEK293T cells expressing WT TRPV3 in the presence or absence agonist Car (300 μM). In the Hoechst 33342/propidium iodide double staining assay, inhibition of NF-κB signaling by potent JSH-23 at 5 μM and 20 μM or inhibition of TRPV3 by ROSA at 20 μM reversed the Car-induced cell death in TRPV3-HEK293T cells (Fig. 8*D*). HEK293T cells expressing TRPV3 in the presence of Car (300 μM) resulted in a 30% ratio of cell death as compared to 8% in the vehicle control group (Fig. 8*E*). In contrast, inhibition of TRPV3 by ROSA at 20 μM or inhibition of the NF-κB signaling pathway by JSH-23 (5 μM and 20 μM) led to significant reduction of cell death ratio to 17%, 21%, and 17%, respectively (Fig. 8*E*). As another positive control, nonspecific TRP channel inhibitor RR (20 μM) also exhibited a reduced cell death ratio to 23% (Fig. 8*E*). In the cell counting kit-8 (CCK-8) cell viability assay, ROSA at 20 μM and JSH-23 at 5 μM or 20 μM reversed the cell death and increased the cell viability rate in HEK293T cells expressing TRPV3 to 95%, 82%, and 94%, respectively, as compared with 72% in the 300 μM Car group (Fig. 8*F*). As a positive control, RR also increased the cell viability rate to 86% (Fig. 8*F*). These results demonstrate that selective inhibition of TRPV3 by ROSA or suppression of NF-κB signaling pathway by JSH-23 reduces cell death induced by TRPV3 agonist Car.

To further confirm the role of downstream NF-κB signaling in dorsal skin inflammation, we topically coapplied TRPV3 agonist Car with the NF-κB signaling inhibitor JSH-23 (20 μM) in 100 μl to mouse dorsal skin for consecutive 4 days and observed a significant alleviation of the dorsal skin inflammation (Fig. 8*G*) and reduction of of dermatitis scores (Fig. 8*H*). These data further demonstrate that blockade of NF-κB signaling pathway by *p*-P65 inhibitor JSH-23 reduces cell death and alleviates skin lesions induced by Car-mediated TRPV3 overactivation.

## Discussion

Natural ROSA possesses potent anti-inflammatory properties and has been implicated in mitigating inflammation associated with conditions such as arthritis, dermatitis, eczema, and acute inflammatory bowel disease (4, 5, 35, 36). ROSA has also been reported to exhibit various other activities including antibacterial, antiviral, and radiation resistance properties (35, 37, 38). As a result, ROSA holds significant practical application value in the fields such as food preservation, feed additives, health products, pharmaceuticals, and cosmetics (39, 40, 41, 42). In this study, we identified the TRPV3 channel as a specific target in the skin for the natural ROSA that is known to alleviate inflammation. At the single-channel level, our results reveal that ROSA can decrease the channel open probability activated by TRPV3 agonists 2-APB and Car without altering the single-channel conductance. Through network pharmacology results, we predicted downstream pathways and verified the NF-κB signaling pathway as downstream of TRPV3 channel through *in vivo* and *in vitro* experiments. Therefore, our findings not only elucidate the TRPV3 in the skin as a direct target of ROSA, providing strong support for the existing development and application of ROSA, but also furnish the developmental potential for therapy of skin diseases.

In addition to ROSA, we have also investigated its analogs CADA and MROSA. Upon its binding, ROSA interacts with residues L635, T636, T665, and F666 in TRPV3, while CADA binds to three residues T636, I637, and T665 and MROSA binds primarily to residue T665. To further verify ROSA and its analogs, we determined the IC_50_ values of CADA and MROSA, which were approximately 3 times and 7 times higher than ROSA, respectively. This finding was also consistent with the scoring results obtained from the molecular docking. Our results from site-directed mutagenesis experiments further elucidated the critical role of residues T636 and T665 in mediating the interactions between the three compounds and TRPV3 channel. Specifically, ROSA interacts with two T636 and two T665 residues from three subunits. In contrast, CADA interacts with two opposite subunits through binding to two T636 and one T665 residues, while MROSA interacts with two adjacent subunits through binding to two T665 residues. Consequently, ROSA occupies the binding pocket with better interactions and demonstrates superior efficacy in inhibiting TRPV3 than CADA and MROSA. The two residues T636 and T665 in TRPV3 correspond to the T599 and T628 residues in TRPV2, although TRPV3 and TRPV2 share relatively low homology of 43% (43), which may explain the weak effect of ROSA on TRPV2. Therefore, subsequent structural modifications of ROSA for achieving better specificity over TRPV2 might need to consider to avoiding targeting the two residues in TRPV2.

The warmth-sensitive Ca^2+^-permeable TRPV3 channel is robustly expressed in the skin and activated by warm temperature and chemical ligands, such as camphor, 2-APB, and skin sensitizer Car. Overactive TRPV3 channels are implicated in various skin diseases including OS, itching, dermatitis, hair growth, and wound healing (20, 22, 43, 44). Consequently, the development of selective TRPV3 channel inhibitors has been explored to address itching, hair loss, and skin inflammation (19, 25, 26, 45, 46, 47). Natural products have been identified as TRPV3 inhibitors, such as forsythoside B, osthole, verbascoside, isochlorogenic acid A and B, and scutellarein (28, 29, 32, 48). Additionally, several synthesized agents, such as a topical anesthetic drug dyclonine and an antispasmodic flopropione, have recently been shown to selectively inhibit TRPV3, reduce itching and alleviate skin inflammation for potential drug repurposing (49, 50, 51). Despite the increasing efforts for discovery of TRPV3 inhibitors, there still remains a need for identification of specific TRPV3 inhibitors.

The NF-κB signaling pathway is a highly conserved across species and a critical cellular mechanism that regulates immune response and cell survival, which is also implicated in various inflammation processes, including UVB injury, and TNF-α or Car-induced inflammation (8, 46, 52, 53, 54, 55, 56). Numerous studies have demonstrated the involvement of the NF-κB pathway in the anti-inflammatory effects of ROSA. ROSA has been shown to downregulate NF-κB pathway activity, thereby resisting inflammatory damage, decreasing the intracellular level of reactive oxygen species, reducing monocyte adhesion and preventing cell death (8, 53, 57). Given the close association between TRPV3 channelopathy and AD, our finding for the role of ROSA in selective inhibition of TRPV3 has enriched the NF-κB signaling pathway as a downstream regulator of TRPV3, which is involved in regulating Car-induced skin inflammatory responses. Therefore, the inhibition of the NF-κB pathway by either JSH-23 or parthenolide can alleviate skin inflammation or prevent UVB-mediated skin photoaging (56). Our previous observations have demonstrated that TRPV3 is upregulated in UVB-induced skin inflammation, while the NF-κB signaling is activated under UV irradiation, leading to the release of various proinflammatory factors and promoting the proliferation of skin keratinocytes and melanocytes (30, 56). This further underscores the importance of inhibiting the NF-κB pathway in preventing skin photoaging and inflammation. We speculate that inhibiting TRPV3 to prevent skin photoaging and inflammation may also be achieved by downregulating NF-κB signaling pathway. However, further validation is needed to support this hypothesis.

In conclusion, our findings not only unveil a novel role of ROSA in inhibiting TRPV3 and subsequently downregulating NF-κB pathway activity but also provide new tools and guidance for further investigations into the pharmacological function of TRPV3 channels. Additionally, our results also confirm that ROSA-mediated inhibition of TRPV3 leads to the downregulation of NF-κB pathway activity, thereby offering further mechanistic insight into the anti-inflammatory activity of ROSA and its analogs in alleviating skin inflammation.

## Materials and methods

### Animals

Adult C57BL/6J mice (aged 6–8 weeks, weighing 20 ± 2 g) were purchased from the Beijing Vital River Laboratory Animal Technology Co. Ltd. TRPV3-KO mice were purchased from Cyagen Biosciences Inc. All mice were housed in plastic cages with natural poplar wood bedding materials, accommodating three to five mice per cage. The housing environment was maintained at a controlled room temperature of (23 ± 2) °C and humidity of (50 ± 5) %, with 12-h light/dark cycle (lights on at 7:00 AM and off at 7:00 PM). Mice had free access to food and water. All animal test protocols were approved by the Institutional Animal Care and Use Committee of Qingdao University Health Science Center and were conducted in accordance with institutional and national guidelines for the use and care of animals in experiments. All mice were anesthetized with isoflurane (1%–1.5%) using an isoflurane gas anesthesia device and were euthanized under anesthesia.

### Compounds and reagents

ROSA (molecular weight [MW]: 360.31), NF-κB inhibitor JSH-23 (JSH-23, MW: 240.34), MROSA, MW: 374.4), CADA (MW: 315.32), and CCK-8 were purchased from Shanghai Tauto Biotech Corporation, Ltd. Compounds 2-APB, A-967079 (A-96), allyl isothiocyanate, Car, capsaicin, menthol, RR, and GSK1016790A (GSK101) were purchased from Sigma-Aldric. All compounds with 99% purity were formulated as stock solutions dissolved in dimethyl sulfoxide. For patch-clamp recordings, compounds were diluted in perfusion solution. For cell death assays, compounds were diluted in cell culture medium. For generation of ear swelling and chronic pruritus models, skin sensitizer Car was used and diluted in 30% ethanol (ethanol/saline = 3/7, v/v). TRPV3, TNF-α, IL-6 and secondary antibodies were purchased from Abcam Shanghai Trading Co., Ltd. P65 and *p*-P65 were purchased from Cell Signaling Technology, Inc.

### Cell culture and plasmid transfection

HEK293T cell line was obtained from the Cell Resource Center, Peking Union Medical College. HEK293T cells were maintained in Dulbecco’s modified Eagle’s medium (DMEM, Gibco) supplemented with 10% fetal bovine serum at 37 °C with 5% CO_2_. HEK293T cells were seeded onto glass coverslips 1 day in advance and transiently transfected with 2.5 mg complementary deoxyribonucleic acids of human TRPV3 (hTRPV3, accession number NM_145068.4), hTRPV1 (accession number NM_080704.3), mTRPV2 (accession number NM_011706.2), hTRPV4 (accession number NM_021625.5), hTRPA1 (accession number NM_007332.3), and hTRPM8 (accession number NM_024080.5) through Lipofectamine 2000 (Invitrogen) following the manufacture’s protocol. The next day, determine the plasmid transfection status by observing green fluorescence based on expression of enhanced GFP as an indicator. All WT or mutant hTRPV3 complementary deoxyribonucleic acid plasmids were confirmed by DNA sequencing. When conducting experiments on HEK293T cells transfected with G573S point mutant TRPV3 plasmid, we replaced culture medium with or without compounds after 4 h of transfection and then observed green fluorescence of HEK293T cell after 10 h. As for the relative fluorescence intensity, we convert the image to 8 bit grayscale using ImageJ (Https://imagej.net/software/fiji/downloads) and set an appropriate threshold value to reduce background noise and enhance the image contrast.

### Electrophysiological recordings

All patch-clamp recordings were conducted 16 to 24 h after transfection using an EPC10 amplifier powered by PatchMaster software (HEKA) (https://www.heka.com/downloads/downloads_main.html#down_patchmaster) at room temperature. The borosilicate glass pipettes were pulled using a DMZ universal electrode puller (Zeitz-Instruments GmbH) to achieve the resistances of three to 5 MΩ (for whole-cell recordings) or five to 9 MΩ (for inside-out patch recordings). Currents were recorded at a potential ramp from −100 mV to +100 mV for 1000 ms with 1000-ms intervals (for whole-cell recordings) or a persistent potential of −60 mV (inside-out patch recordings). Both pipette solution and bath solution contained (in mM): 130 NaCl, 3 Hepes, and 0.2 EDTA (pH = 7.2–7.4). All recording data were analyzed using Igor Pro (Wave-metrics) (https://www.wavemetrics.com/order/order_igordownloads.htm) and Origin 8.6 (Origin Lab) (https://www.originlab.com/doc/zh/Quick-Help/Download-Installer).

### Molecular docking

Molecular docking was conducted using Schrödinger Glide (Maestro software suite 2019, Schrödinger) to dock ROSA, CADA, and MROSA to the cryo-EM mTRPV3 structure (PDB ID code: 6DVW). This was based on the observation that two ROSA analogs, dicaffeoylquinic acid isomers isochlorogenic acid A and isochlorogenic acid B, were confined within the channel central cavity pocket near the pore helix and S6 segment (28). Chemical structures of ROSA and its analogs were drawn using ChemDraw (CambridgeSoft). The compounds and docking models were semiflexibly docked using the built-in program Ligprep after optimization through energy minimization.

### Network pharmacology

Three online databases including PUBCHEM (https://pubchem.ncbi.nlm.nih.gov), TCSMP (https://old.tcmsp-e.com/tcmsp.php), and ChEMBL (https://www.ebi.ac.uk/chembldb/) were used to predict possible targets for ROSA effects. The GeneCards database was used to predict possible targets for TRPV3-associated OS and AD. Kyoto Encyclopedia of Genes and Genomes pathway analysis was used to identify potential signaling pathways, and the analysis was conducted using DAVID, a web server for functional enrichment analysis and functional annotation of gene lists, and the data visualization was conducted using Hiplot Pro (https://hiplot.com.cn/), a comprehensive web service for biomedical data analysis and visualization (58, 59).

### Cell death and proliferation assay

For cell death assay, HEK293T cells transfected with hTRPV3 were seeded in 6-well plates and randomly divided into different groups. TRPV3 and NF-κB inhibitors were added 1 h before the addition of Car. After administration of Car for 1 h, cell death was detected using Hoechst 33342/propidium iodide Double Stain Kit (Solarbio). After staining, cells were resuspended with PBS buffer. Five random view images in each group were taken under a microscope (Eclipse Ti, Nikon) and counted using ImageJ software (National Institutes of Health).

For cell viability assay, hTRPV3-HEK293T cells were seeded in 96-well plates for 12 to 16 h before exposed to Car in DMEM containing 2% fetal bovine serum for 1 h. TRPV3 and NF-κB inhibitors were added 1 h before the addition of Car. After removing the medium containing chemical compounds, hTRPV3-HEK293T cells were incubated with DMEM containing 10% CCK-8 (TargetMol) for 1 h. Cell viability was then assessed using the Sunrise microplate reader under the light absorbance at 450 nm (TECAN, CH).

### Models of ear swelling and dermatitis induced by skin sensitizer Car

Skin sensitizer Car was dissolved in saline solution containing 30% ethanol. The dorsal skin or ear of mice was swabbed with 3% Car for four consecutive days. To investigate the effects of ROSA, it was diluted to concentration of 0.1/1/10 mM with saline and topically applied on the ear and dorsal skin once a day for 4 days. The control group only received swabs with the solvent.

### Western blot

Skin or ear skin tissues were homogenized in radioimmunoprecipitation assay buffer (Thermo Fisher Scientific) containing protease inhibitors at a ratio of 100:1 per 10 mg tissue and centrifuged at 15,000 rpm for 15 min. The protein concentration was quantified using a bicinchoninic acid kit (Thermo Fisher Scientific), followed by SDS-PAGE for Western blot analysis. Protein transfer was performed at 150 mA for 90 min, and the transferred membranes (Millipore) were incubated with primary antibodies (TRPV3 antibody, 1:1000, TNF-α antibody, 1:1000, IL-6 antibody, 1:2500, P65 antibody, 1:1000 and *p*-P65 antibody, 1:1000) in 5% skim milk (Becton, Dickinson and Company). Subsequently, membranes were incubated with secondary antibodies (goat anti-mouse and goat anti-rabbit, Bioss, 1: 10,000) at room temperature for 1 h or at 4 °C for 6 to 8 h before visualization by the ECL system (Thermo Fisher Scientific). Images were quantified using the Image Lab software (https://www.bio-rad.com/zh-cn/product/image-lab-software?ID=KRE6P5E8Z).

## Statistical analysis

All data were expressed as the mean ± SD. Statistical significance was performed using *t* test, one-way, and two-way ANOVA followed by the indicated multiple-comparison test, using GraphPad Prism 7.0 software (GraphPad Software) (https://www.graphpad.com/rf/8500902037/). A value of *p* < 0.05 was considered statistically significant. The Spearman rank correlation coefficient method was used to investigate the correlation between the severity of ear skin inflammation and ear swelling scores.

## Ethics approval

All animal experimental procedures were approved by the Experimental Animal Ethics Committee of Qingdao University College of Medicine and were performed under the guidelines of the Bylaw of Experiments on Animals.

## Data availability

The authors declare that all the presented data supporting the findings of this study are contained within this article.

## Conflict of interest

The authors declare that they have no conflicts of interest with the contents of this article.
